# Performing Antegrade Selective Cerebral Perfusion Using the AV Cannula: A Novel Approach

**DOI:** 10.1055/s-0039-3401997

**Published:** 2020-02-10

**Authors:** Andrea Venturini, Alan Gallingani, Angiolino Asta, Chiara Zanchettin, Giampaolo Zoffoli, Antonio Cannarella, Domenico Mangino

**Affiliations:** 1 Department of Cardiac Surgery, Ospedale dell'Angelo, Mestre, Venezia, Italy

**Keywords:** cerebral protection, aortic arch, aortic dissection, antegrade cerebral perfusion

## Abstract

Antegrade selective cerebral perfusion has become the preferred choice for brain protection during aortic arch surgery. To perform antegrade selective cerebral perfusion, cannulas have been introduced directly into the ostia of the supra-aortic vessels (SAV) after institution of hypothermic circulatory arrest and opening the aortic arch. We describe a different surgical technique with a new type of cannula for antegrade selective cerebral perfusion. This cannula, called AV (Andrea Venturini) cannula, has been designed to be introduced in the SAV directly using a standard guidewire technique (Seldinger's technique). The AV cannula can also be introduced from the ostia of the SAV if preferred. The AV cannula can be introduced before the institution of hypothermic circulatory arrest and before opening the aortic arch. One great advantage of this technique is that the ostia of the SAV remain free from a cannula, allowing the operator easier access and a faster anastomosis or reimplantation.

## Introduction

Brain protection is one of the crucial issues in aortic arch surgery. Possible techniques include deep hypothermic circulatory arrest, retrograde cerebral perfusion, or the combination of both. 1 2

Following the first publications by Profs. Kazui et al and Bachet et al, 3 4 5 antegrade selective cerebral perfusion (ASCP) has become the preferred choice in most institutions.

Because the cannula in the original technique had to be placed through the ostia of the supra-aortic vessel (SAV), it needed hypothermic circulatory arrest (HCA) and opening of the aortic arch before instituting ASCP. The brain was not protected during this initial period (opening of the arch), although that is usually very short, in some cases can take a few minutes or substantially more in redo procedures.

The presence of the cannula in the SAV ostia can also get in the way of the operator leading to a more demanding anastomosis with longer circulatory arrest times.

The cannulas also have to be removed before the completion of the anastomosis, exposing the brain to further unprotected ischemic time.

We describe a new surgical technique with the AV (Andrea Venturini) cannula (Med Europe SRL, Bologna, Italy) that is introduced directly into the SAV vessel so that the brain perfusion can be commenced immediately before HCA, freeing the SAV ostia for easier access and reimplantation.

## Methods

**Fig. 1:**
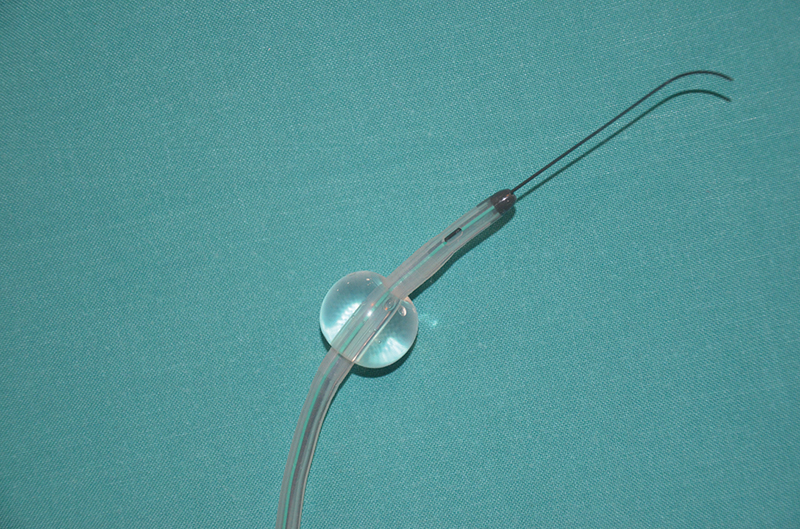
AV cannula with a guidewire. AV, Andrea Venturini.

AV cannula is a silicone cannula with a malleable steel shaft. It has been specifically designed to be introduced directly into SAV and not just from the ostia of the vessel. This cannula is similar to existing cannulas with an inflatable balloon at the tip to seal the vessel proximally while perfusing distally ( Fig. 1 ).

The novelty of this device is the possibility of a guidewire introduction using the Seldinger's technique. 6

After median sternotomy and institution of cardiopulmonary bypass, SAV are isolated and encircled with an umbilical tape. We routinely ligate the innominate vein only during total arch replacement surgery.

A 5/0 prolene (possibly pledget reinforced) purse string suture is placed on the innominate artery and on the left common carotid artery wall. The vessel is then punctured with a specific needle and a guidewire is introduced a few centimeters into the lumen of the vessel. If required, before introducing the cannula a small incision with a number 11 blade can also be done on the vessel wall. If necessary, standard available vessel introducing dilators can be used to facilitate this maneuver.

**Fig. 2:**
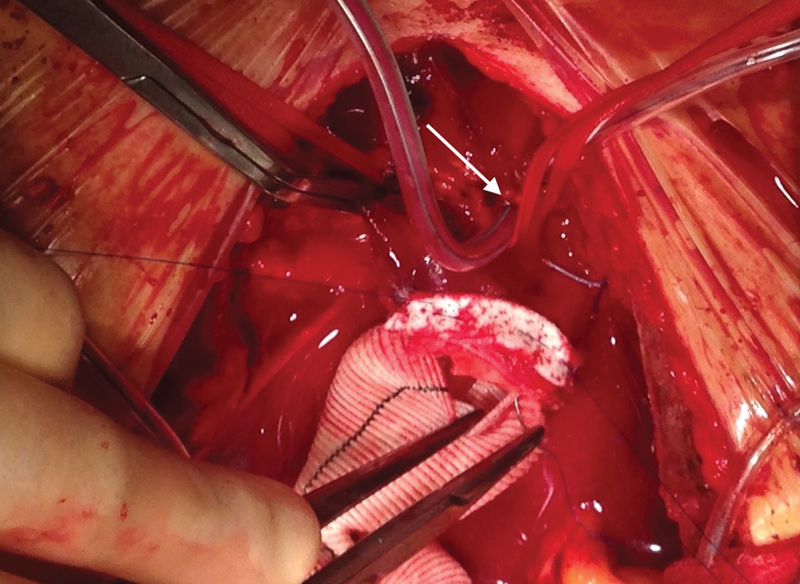
Andrea Venturini cannula is introduced in to the left common carotid artery (white arrow) during hemiarch operation. A clamp is on the innominate artery for right brain perfusion through a right axillary artery cannula.

The AV cannula is then inserted into the innominate artery and in the left common carotid artery and a purse string suture is tied and secured ( Fig. 2 ).

If required the left subclavian artery can also be cannulated with the same devices and technique.

As systemic cardiopulmonary bypass is discontinued, ASCP is started with a flow of 10 to 15 mL/kg/min maintaining NIRS (Near Infrared Spectroscopy) values between 50 and 80% rsO2, as described in the literature. 7 8 9 10 Blood pressure from the cannula itself can also be monitored.

The required arch operation 11 is then performed and only after restarting full-flow cardiopulmonary bypass the AV cannulas can be removed.

## Results

We operated two patients so far using the AV cannula. The first one was a 67-year-old male who presented with a typical Type A acute aortic dissection without involvement of the SAV. We performed replacement of the ascending aorta and the proximal arch (hemiarch operation). The second patient was a 74-year-old male with a contained rupture of the dissected proximal descending thoracic aorta and severe mitral valve regurgitation. He previously underwent ascending aorta replacement for acute Type A dissection, subsequently aortic root replacement 1 year later. We performed a total arch replacement with the frozen elephant trunk technique using a Thoraflex Hybrid prosthesis (Vascutek Terumo, Scotland, United Kingdom) and mitral valve replacement using St. Jude Medical mechanical prosthesis (St. Paul, MN).

In the first patient, cardiopulmonary bypass time was 97 minutes, aortic crossclamp time was 84 minutes, circulatory arrest and selective cerebral antegrade perfusion times were 22 minutes; whereas in the second patient, the times were 246, 153, 44, and 95 minutes, respectively.

No patient required rethoracotomy for bleeding; furthermore, no stroke and spinal cord injury were detected. At the moment, both intraoperatively and at CT scan follow-up no significant stenosis of the cannulation sites were noted. Follow-up at 6 months found that both patients are alive and free of new onset major neurological events.

## Conclusion

Transarterial introduction using Seldinger's technique of the AV cannula represents an alternative to the current well-established techniques.

**Fig. 3:**
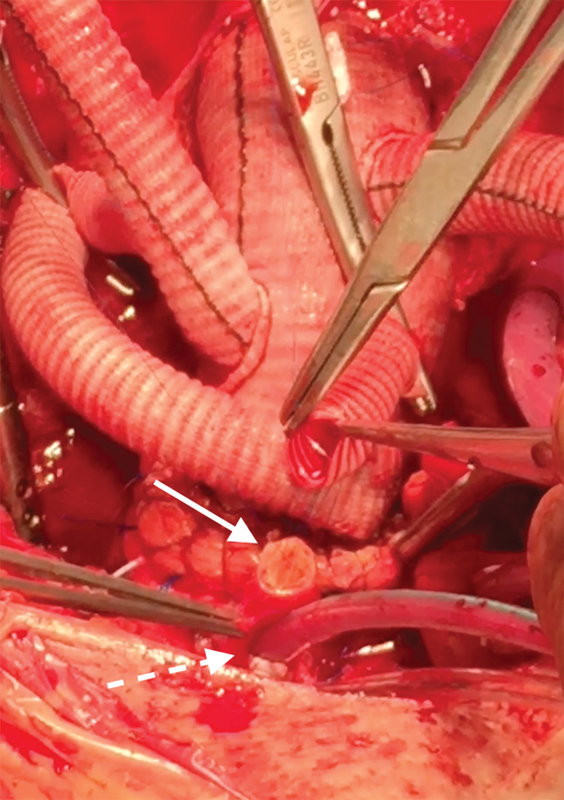
Left internal carotid artery anastomosis. Free anastomotic site indicated by white arrow; AV cannula insertion site indicated by dashed white arrow.

The major advantages of the technique we describe are complete cerebral protection throughout the HCA time and easier arch vessel reimplantation or hemiarch operations as the AV cannula is out of the way of the surgeon ( Figs. 3 4 5 6 ).

**Fig. 4:**
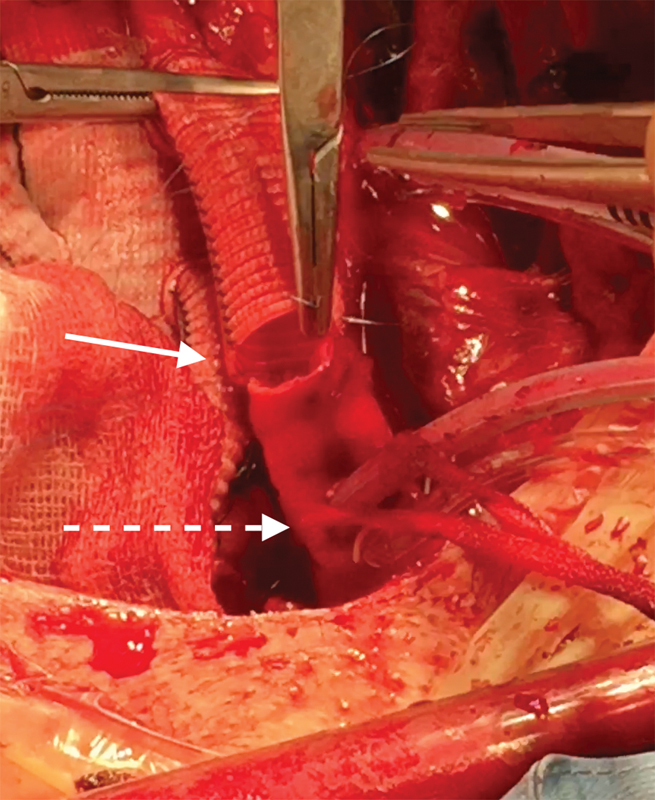
Innominate artery anastomosis. Free anastomotic site indicated by white arrow; Andrea Venturini cannula insertion site indicated by dashed white arrow.

**Fig. 5:**
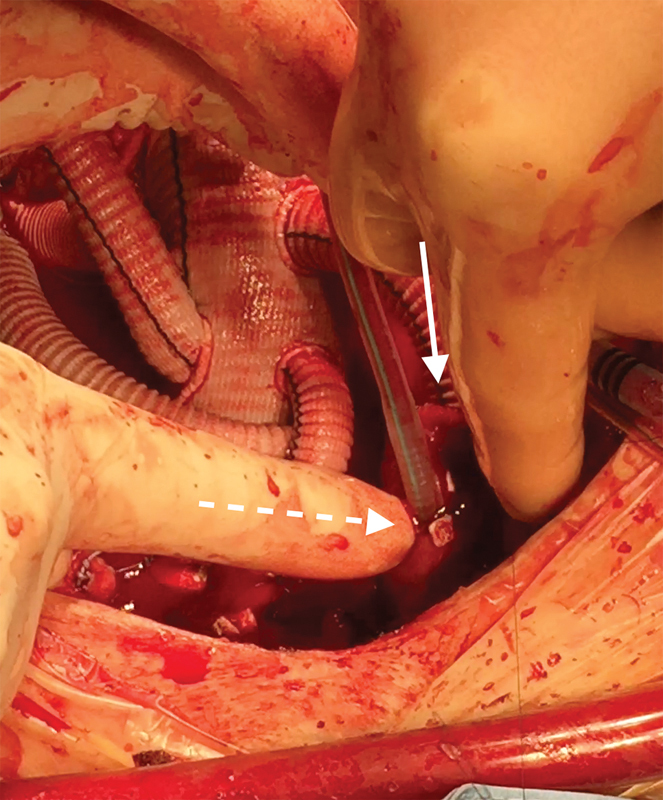
Andrea Venturini (AV) Cannula removal. Dashed white arrow: easy AV cannula removal, thanks to enough distance from anastomosis line (white arrow).

**Fig. 6:**
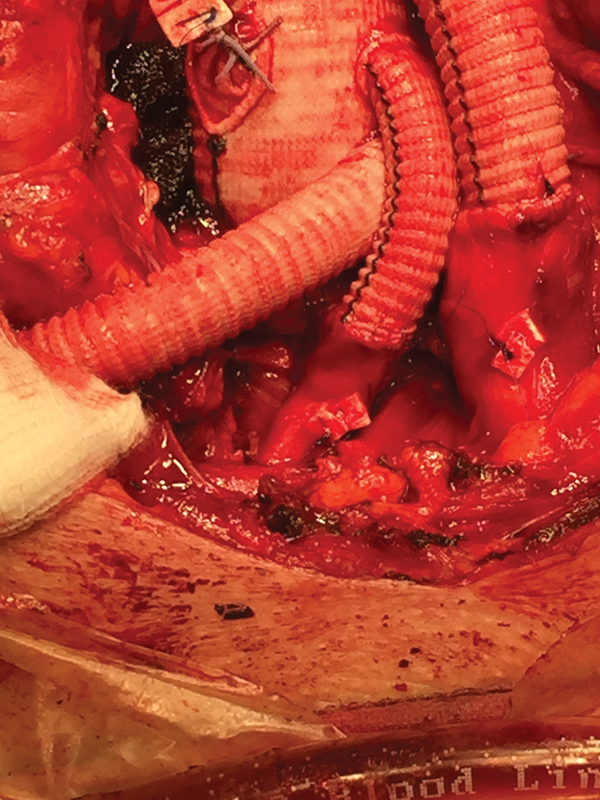
Supra-aortic vessels detail after frozen elephant trunk operation.

The only controindication at present is the presence of dissection of the SAV vessel itself. In this situation, the traditional technique remains the preferred option.

The AV cannula technique for ASCP could possibly be a safer option for instituting cerebral perfusion during HCA in selected patients; in many situations, it is a faster and easier alternative to the existing surgical technique.

## Notes

Editor's Questions

1. *Is this new aortic branch vessel perfusion cannula approved for use in Europe? In the United States?*

The new AV cannula has obtained approval for use in Europe, but not yet in the United States.
